# Invasive plant richness and abundance synergistically amplify co-invasion success: Experimental evidence for invasional meltdown

**DOI:** 10.1016/j.pld.2026.03.004

**Published:** 2026-03-11

**Authors:** Yi Hu, Xiao Guo, Zhenwei Xu, Jingfeng Wang, Ruiqi Sun, Mingyan Li, Pengcheng Zhu, Karin M. Kettenring, Hana Skálová, Weihua Guo

**Affiliations:** a Qingdao Key Laboratory of Ecological Protection and Restoration, School of Life Sciences, Shandong University, 72 Binhai Road, Qingdao 266237, China; b College of Landscape Architecture and Forestry, Qingdao Agricultural University, Qingdao 266109, China; c Department of Watershed Sciences, Utah State University, Quinney College of Natural Resources, Logan, UT, United States; d Department of Invasion Ecology, Institute of Botany of the Czech Academy of Sciences, Průhonice 25243, Czech Republic

**Keywords:** Interactions among invasive plant species, Invasion success, Propagule pressure, Plant invasion, Selection effect

## Abstract

Co-invasion by multiple invasive species is becoming increasingly prevalent, yet the role of interactions among invasive species in shaping invasion success remains poorly understood. While invader abundance is known to facilitate establishment, the combined effects of invasive plant richness and abundance on invasion outcomes have rarely been tested experimentally. We established a fully factorial mesocosm experiment manipulating four levels of invasive plant richness (0, 1, 2, or 3 species) and abundance (0, 12, 24, 48 individuals) using three Asteraceae invaders—*Erigeron canadensis*, *Erigeron annuus*, and *Bidens pilosa*—within native plant communities. We found facilitative and neutral interactions among invaders generated non-additive (reversal) negative effects on native biomass, which intensified with increasing invader richness. Higher richness facilitated invasion success by modifying selection effects among invasive species—primarily through size-mediated mechanisms—that enhanced invasive biomass and strengthened suppression of native plants. Invasive richness altered soil nitrogen pools without promoting invasion success, but was associated with changes in soil microbial biomass carbon and litter decomposition that coincided with reduced native performance. Elevated invader abundance further promoted invasion success and interacted synergistically with richness to amplify invasion impacts, indicating that co-invasion severity may intensify under future scenarios of increasing propagule pressure. Overall, our findings provide experimental evidence for the invasional meltdown hypothesis and demonstrate how invasive plant richness and abundance jointly enhance invasion success. Our results further suggest that early management targeting low-abundance invaders may disrupt the synergistic effect between invasive plant richness and abundance and mitigate the impacts of multispecies co-invasion.

## Highlights

• Positive and neutral interactions of invasive species produce non-additive effects.
• Invader richness boosts invasive biomass and suppresses natives via selection effects.
• Selection effects are primarily driven by size-mediated mechanisms.
• Invasive plant abundance synergizes with richness to amplify invasion success.

## 1 Introduction

Native plant communities worldwide are increasingly threatened by multiple invasive plant species originating from diverse biogeographic regions (Pyšek et al., 2020; Seebens et al., 2021). These co-invasions jeopardize native biodiversity (Liu et al., 2025), disrupt ecosystem functioning (Peller and Altermatt, 2024), and impose substantial economic costs (Diagne et al., 2021; Soto et al., 2025). While interactions between invasive and native species are widely recognized as key determinants of invasion success (Hu et al., 2022; Xiang et al., 2024; Jin et al., 2025), growing evidence suggests that interactions among invasive species themselves can also play a crucial role (Kuebbing and Nunez, 2015; Ren et al., 2023; Sun et al., 2024). The number of co-invading species continues to rise and has not yet reached saturation (Seebens et al., 2017; Capinha et al., 2023; Liu et al., 2024; Roy et al., 2024). Nevertheless, most empirical research has focused on interaction between single invaders and native communities or, less commonly, on pairwise interactions between two invaders (Kuebbing et al., 2013; Guo et al., 2023a; Wei and van Kleunen, 2025). Studies explicitly addressing the interactions among multiple invaders remain scarce. Yet when several invaders establish simultaneously, their combined effects on invasion success and native communities may differ substantially from those expected under single-species invasion scenarios (Brandt et al., 2023; Ahmad et al., 2025; Shen et al., 2026). Understanding the mechanisms driving these dynamics is therefore critical for predicting invasion outcomes in multi-species contexts.

The direction and strength of interactions among co-occurring invaders represent a critical but understudied aspect of co-invasion dynamics. According to the invasional meltdown hypothesis, positive interactions among invasive species can reinforce establishment and spread, thereby magnifying impacts on native communities (Simberloff and Von Holle, 1999; Braga et al., 2018; Yin et al., 2022). Conversely, invasional interference occurs when invaders inhibit one another through competition (Rauschert and Shea, 2012; Lenda et al., 2019; Oduor et al., 2024). Such positive or negative interactions among invasive species frequently lead to non-additive effects on native communities, where the combined impact of multiple invaders differs from the sum of their individual effects observed in monocultures (Braga et al., 2020; Brandt et al., 2023). In contrast, neutral interactions result in additive outcomes (Kuebbing and Nunez, 2015; Tekiela and Barney, 2017). Although the overall impact of multiple invaders on native communities is typically negative, the magnitude of suppression varies depending on whether interactions are facilitative, antagonistic, or neutral (Kuebbing and Nunez, 2016). Therefore, determining whether interactions among invaders produce additive or non-additive effects on the growth of native species is fundamental for elucidating the mechanisms govern multi-species co-invasion.

Diversity effects (complementarity and selection effects) provide a conceptual framework for understanding how multiple species interact to influence community productivity (Loreau and Hector, 2001; Xu et al., 2024; Chen et al., 2025). Complementarity effects arise from niche differentiation or facilitation, enhancing resource-use efficiency and mutual growth (Hooper et al., 2005; Barry et al., 2019; Godoy et al., 2020; Feng et al., 2022). Selection effects, in contrast, occur when highly productive or competitive species dominate and contribute more to total biomass than expected from their initial abundance (Wilsey et al., 2009; Wang et al., 2024; Xu et al., 2025). However, when chemical or physical interference occurs among co-occurring plants, antagonistic interactions may suppress biomass in mixtures relative to monocultures, reflecting negative complementarity (Loreau, 1998; Polley et al., 2003). A negative selection effect emerges when low-productivity species, rather than high-productivity ones in monoculture, dominate the community (Loreau and Hector, 2001; Wang et al., 2025b), potentially reducing overall community productivity (Loreau, 2000). Therefore, complementarity and selection effects among invasive species may also influence invasive biomass. Given that invasive species typically possess superior competitive traits compared with natives and often suppress native plant growth (Ordonez et al., 2010; van Kleunen et al., 2010; Xiang et al., 2024), these diversity effects could jointly promote invasion success. However, empirical evidence remains scarce: only one study to date has demonstrated a role of selection effects in facilitating invasion success (Wang et al., 2022), while the broader contributions of diversity effects to co-invasion remain poorly tested. Furthermore, Tatsumi and Loreau (2023) proposed a method for partitioning biodiversity effects into density and size components, providing a framework to assess changes in survival and individual growth. However, this approach has not yet been applied to multiple plant invasions.

A further factor shaping invasion success is abundance (propagule pressure), which enhances fitness or the probability of establishment and persistence (Lockwood et al., 2005; Simberloff, 2009). High propagule pressure can enable invaders to overcome biotic resistance from native communities (Von Holle and Simberloff, 2005; Cassey et al., 2018; Murillo and Wagner, 2025) and is a well-recognized driver of single-species invasion success (Liu et al., 2022; Rojas-Botero et al., 2022). Interactions among invasive species may also depend on their abundance (Zhang and Tielbörger, 2020). When multiple invaders co-occur, increasing abundance may enhance resource utilization efficiency (MacDougall et al., 2009; Sun et al., 2024), thereby strengthening complementarity effects and amplifying facilitative interactions among invaders. Alternatively, greater abundance may promote the dominance of highly competitive invasive species (Stringham and Lockwood, 2021; Wang et al., 2022), leading to stronger selection effects. Together, these processes can intensify the suppression on native species through competitive exclusion and ultimately facilitating invasion success (Hart, 2023). Conversely, excessive invasive plant abundance may increase niche overlap and intensify interspecific competition among invaders for limited resources, weakening complementarity effects and potentially reducing invasion success (Adler et al., 2018; Sardans et al., 2021). Despite these plausible mechanisms, empirical evidence for the interactive effects between invasive plant richness and abundance on invasion success remains scarce and inconclusive, necessitating further investigation.

Here, we conducted a mesocosm experiment using a full factorial design manipulating invasive plant richness and abundance, which were introduced into established native communities. We quantified invasion success as the proportion of invasive biomass, while also measuring native biomass to assess impacts on resident communities. We further assessed the direction and magnitude of interactions among invaders and tested whether their effects on natives were additive or non-additive. Using an additive partitioning framework (Loreau and Hector, 2001; Chen et al., 2025), we evaluated the contributions of complementarity and selection effects among invasive species to invasion success. Because soil microbial activity and nutrient dynamics can mediate plant competition and invasion outcomes (Guo et al., 2023b; Zhang et al., 2023), we further examined how invasive plant richness and abundance affect soil biochemical properties to identify potential belowground mechanisms of invasion success.

Specifically, our study addressed three key questions: (1) Are interactions among invasive species positive or negative, and are their impacts on native community additive or non-additive? (2) Does invasive plant richness enhance invasion success, and if so, do complementarity or selection effects underpin this relationship? How do density-mediated and size-mediated complementarity and selection effects respond? (3) Does invasive plant abundance modify the effects of richness on invasion success?

## 2 Materials and methods

### 2.1 Study species

The Asteraceae family contains the greatest number of naturalized and invasive taxa (Pyšek et al., 2017; Yang et al., 2025). We selected three highly invasive and widely distributed species—*Erigeron canadensis*, *Erigeron annuus* and *Bidens pilosa*—all classified as aggressive invaders (Hao and Ma, 2023). These species exhibit broad ecological tolerance, enabling them to colonize a wide range of habitats. They also release allelochemicals that inhibit neighboring vegetation (Lu et al., 2020; Balah et al., 2024), frequently leading to dominance and co-occurrence in field ecosystems (Chen et al., 2013). Seedlings of the three invaders were collected from natural populations adjacent to the experimental greenhouse.

The native community was assembled from eight dominant and ecologically important species characteristic of the Yellow River Delta: *Suaeda glauca*, *S*. *salsa*, *Apocynum venetum*, *Limonium bicolor*, *Artemisia capillaris*, *Glycine soja*, *Phragmites australis*, and *Setaria viridis* (Table S1). Seed materials were obtained from the Yellow River Delta Field Experiment Station (37°51′9″ N, 118°48′53″ E) in October 2020 and stored at 4 °C under controlled humidity until use.

### 2.2 Greenhouse setup and experimental design

A greenhouse mesocosm experiment was implemented from June to November 2021 in Majiabaimiao Village, Qingdao, China (36°22′54″ N, 120°36′5″ E). Native seedlings were first raised in May 2021 in plastic containers (52 × 33 × 15 cm) filled with a 1:2 mixture of peat and river sand (6 L:12 L). In June 2021, 48 individuals (six of each native species) of ∼10 cm height were transplanted into the containers filled with the same substrate as used for seedling cultivation. They were arranged in a randomized 6 × 8 matrix to ensure consistent planting density and spatial organization across all mesocosms. During the two-week establishment phase, any dead native individuals were replaced to ensure consistent initial density. Once native communities were established, invasive plants (∼20 cm tall) were introduced and evenly distributed into the spaces between native plants to establish richness–abundance treatments (Fig. S1). Within two weeks following the introduction of invaders, any dead invasive individuals were also replanted to maintain target abundance levels.

**Fig. 1:**
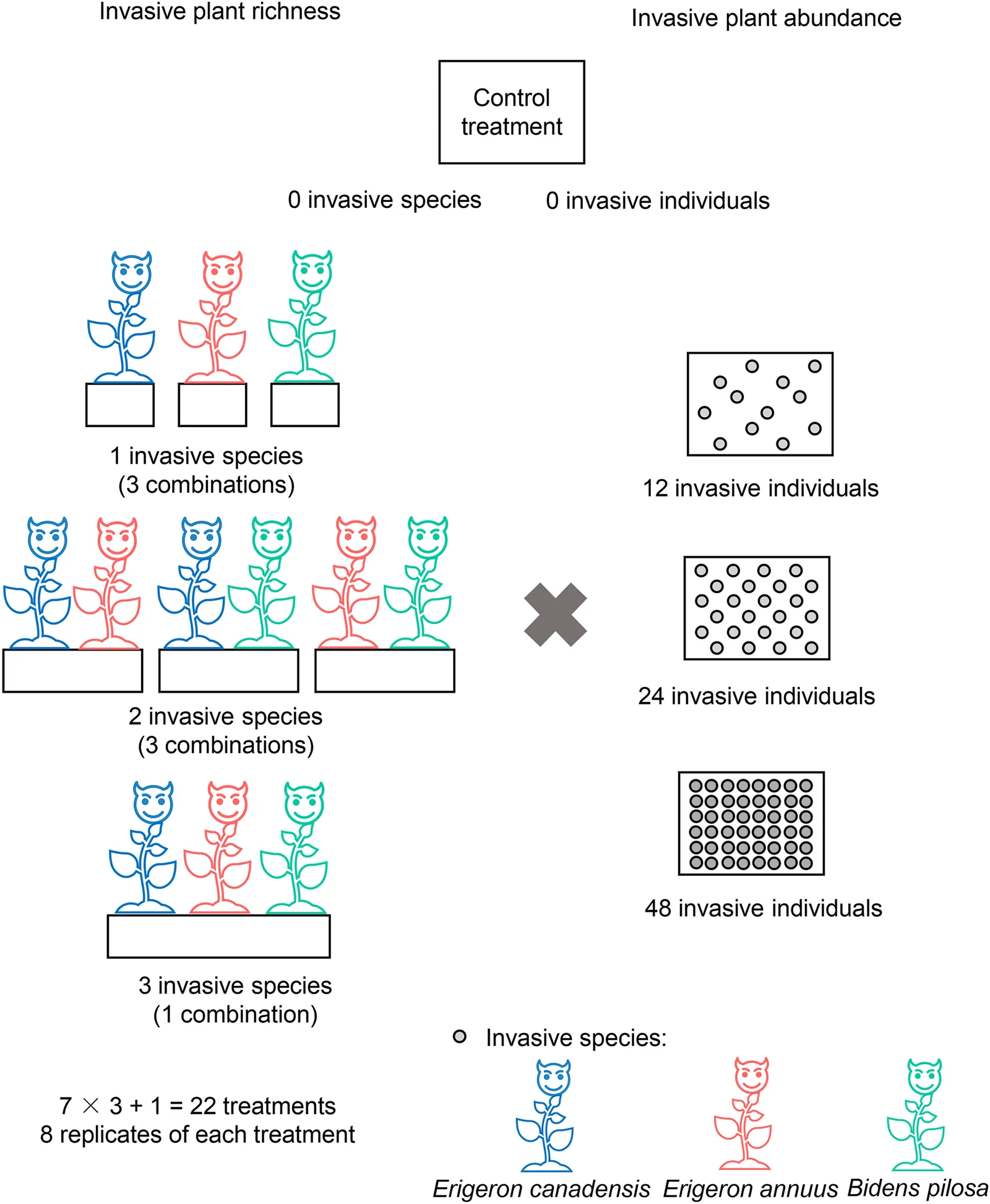
Experimental design schematic. This study implemented crossed gradients of invasive plant richness (0, 1, 2, or 3 species) and abundance (0, 12, 24, or 48 individuals) in mesocosms. The control treatment (0 richness/abundance) contained only native plants. Invasive plant richness levels consisted of seven combinations of three Asteraceae species: *Erigeron canadensis* (blue), *Erigeron annuus* (red), and *Bidens pilosa* (green). Grey circles represent invasive individuals, with their number indicating abundance level. Details of planting patterns are provided in Fig. S1.

The fully factorial design included four levels of invasive richness (0, 1, 2, 3 species) crossed with four abundance levels (0, 12, 24, 48 individuals per container). Invasive plant abundance referred to the total number of invasive individuals per container, and when multiple invasive species co-occurred, individuals of each invasive species were planted in equal numbers. To evaluate the effects of invasive species on native communities, abundance treatments were established under an additive design, maintaining 48 native individuals constant across all mesocosms. This design simulated a gradient of invasion intensity, ranging from early colonization (low abundance) to high propagule pressure (high abundance). The control treatments consisted of native communities without invaders (richness = 0, abundance = 0). Invasive plant richness treatments comprised all possible combinations of the three Asteraceae invaders (Schematic: Fig. 1; Full treatment matrix: Table S2): monocultures (*E*. *canadensis*, *E. annuus*, *B*. *pilosa*), two-species mixtures (*E. canadensis*–*E. annuus*, *E. canadensis*–*B. pilosa*, *E. annuus*–*B. pilosa*), and three-species polyculture (*E. canadensis*–*E. annuus*–*B. pilosa*). Each treatment was replicated eight times, yielding 176 mesocosms in total. To minimize spatial heterogeneity, we implemented randomized blocking across all 176 mesocosms.

### 2.3 Sampling and measurements

Above-ground biomass was harvested in November 2021 by clipping all vegetation at the soil surface and the surviving invasive individuals (realised abundance) were counted. Samples were sorted by species, oven-dried at 80 °C for ≥ 48 h, and weighed to constant mass. The total above-ground biomass was divided into invasive and native above-ground biomass. We then calculated invasion success as the proportion of invasive above-ground biomass relative to total above-ground biomass. In the following text, above-ground biomass is referred to simply as “biomass”.

To examine how invasive plant richness and abundance affect soil properties and microbial activity, and to determine whether changes in these belowground factors mediate invasion outcomes, we measured soil enzyme activities (β-glucosaminidase, β-glucosidase, and acid phosphatase), microbial biomass carbon, ammonium nitrogen (NH_4_^+^–N), nitrate nitrogen (NO_3_^-^–N), total nitrogen, total phosphorus, organic carbon, and litter decomposition. Soil was sampled twice during the experiment. The first sampling, conducted at the vigorous growth stage (September 2021), aimed to assess peak microbial activity and nitrogen availability. A composite soil sample per mesocosm was collected using the five-point method (five soil cores combined into one sample; core diameter 4 cm, depth 0–5 cm). Fresh samples were sieved (1 mm) and used to determine soil enzyme activities, microbial biomass carbon, NH_4_^+^–N, and NO_3_^-^–N. The second sampling, performed at harvest (November 2021), was designed to capture the overall effects of invasive plant richness and abundance on soil stocks throughout the growing period. These samples were air-dried and sieved (1 mm) for analysis of total nitrogen and total phosphorus, and organic carbon was determined after sieving through 0.2 mm mesh. In total, 352 soil samples were collected across both sampling periods. Litter decomposition rates were estimated using reed litterbags. The Supplementary Information provides full measurement details.

### 2.4 Quantifying invasive species interactions

To determine whether the interactions among invasive plant species were positive or negative, we calculated biomass deviation of invasive plant species (D_i_) according to the formula (Loreau, 1998):

(1) $$D_{i}=\left(O_{i}\;-\;E_{i}\right)\;/\;E_{i}$$

where O_i_ is the observed invasive biomass in the species mixture (i.e., two- or three-species combinations), while E_i_ is the expected invasive biomass, derived from monoculture yield multiplied by its initial proportion in the mixture. Values of D_i_ > 0 indicate positive interaction among invasive species (facilitation or complementarity), while D_i_ < 0 reflects negative interaction among invasive species (competition).

### 2.5 Quantifying additive vs. non-additive effects on natives

To explore the impacts of invasive plant richness and abundance on native biomass, we calculated the response index, which intuitively reflects both the direction and magnitude of invasion effects relative to the control:

(2) $$\text{Response}\;\text{index}=\ln\left(X_{a,b}\right)–\ln\left(X_{\text{control}}\right)$$

where X_a,b_ represents native biomass in invasive plant richness a and abundance b treatments, X_control_ is native biomass in control treatment.

To determine whether the impacts of the interaction among invasive plant species on native biomass are additive or non-additive, we calculated the difference between the observed and additive expected native biomass (DOE), as follows:

(3) $$\text{DOE}=R_{\text{observed}}\;–\;R_{\text{expected}}$$

where R_observed_ represents the observed response index of native biomass in the mixture, R_expected_ indicates the additive expected response index, the sum of monoculture response index divided by the number of invasive species in the mixture. The monoculture response index was calculated as ln (X_monoculture_) – ln (X_control_). When DOE does not differ significantly from zero, it indicates additive effects, meaning that the combined impact of multiple invaders equals the sum of their individual effects. Positive DOE values denote synergistic non-additive effects, where the joint impact exceeds additive expectations. Conversely, negative DOE values may reflect two scenarios: if the R_observed_ and R_expected_ share the same sign, they indicate antagonistic non-additivity (weaker combined effects than expected); if the signs differ, they represent reversal effects, where the combined invader effect opposes the additive expectation (Ahmad et al., 2025).

### 2.6 Partitioning complementarity and selection effects among invasive species

Complementarity effects reflect yields (above-ground biomass) gain from niche differentiation and facilitation in mixtures exceeding monoculture-based predictions. Selection effects emerge when species with superior monoculture yields dominate mixtures. We applied the additive partitioning method of Loreau and Hector (2001) to estimate net diversity, complementarity, and selection effects among invaders:

(4) $$\text{Net}\;\text{diversity}\;\text{effect}=N\bar{\Delta RY}\bar{M}+Ncov\left(\Delta RY,M\right)$$

(5) $$\text{Complementarity}\;\text{effect}=N\bar{\Delta RY}\bar{M}$$

(6) Selectioneffect=Ncov(ΔRY,M)

where N is the number of invasive species, ΔRY is deviation of observed relative biomass of invasive species from initial planting proportion, *M* is the invasive biomass in monoculture mesocosm, cov(ΔRY,M) is the covariance between *M* and *ΔRY*.

Following Tatsumi and Loreau (2023), we further partitioned complementarity and selection effects into density-mediated and size-mediated components to distinguish whether biodiversity effects arise from differences in survival or growth. Density-mediated effects capture changes in species performance driven by variation in the number of surviving individuals (i.e., plant density, referred to as realized abundance in this study), whereas size-mediated effects reflect changes associated with the average biomass per individual (i.e., plant size). A positive density- or size-mediated complementarity effect indicates that multiple species benefit proportionally through enhanced survival or growth, respectively. In contrast, a positive density- or size-mediated selection effect suggests that species with higher monoculture yields gain disproportionately greater survival or growth advantages in mixtures. Negative values of these components represent the opposite patterns. We calculated density-mediated and size-mediated complementarity and selection effects using *densize* package (Tatsumi and Loreau, 2023):

(7) $$\text{Net}\;\text{diversity}\;\text{effect}=\sum_{i=1}^{N}\frac{\bar{M}}{{2M}_{i}}\left(W_{O,i}+W_{E,i}\right)\Delta D_{i}+\sum_{i=1}^{N}\frac{M_{i}-\bar{M}}{{2M}_{i}}\left(W_{O,i}+W_{E,i}\right)\Delta D_{i}+\sum_{i=1}^{N}\frac{\bar{M}}{{2M}_{i}}\left(D_{O,i}+D_{E,i}\right)\Delta W_{i}+\sum_{i=1}^{N}\frac{M_{i}-\bar{M}}{{2M}_{i}}\left(D_{O,i}+D_{E,i}\right)\Delta W_{i}$$

(8) $$\text{Density}-\text{mediated}\;\text{complementarity}\;\text{effects}=\sum_{i=1}^{N}\frac{\bar{M}}{{2M}_{i}}\left(W_{O,i}+W_{E,i}\right)\Delta D_{i}$$

(9) $$\text{Density}-\text{mediated}\;\text{selection}\;\text{effects}=\sum_{i=1}^{N}\frac{M_{i}-\bar{M}}{{2M}_{i}}\left(W_{O,i}+W_{E,i}\right)\Delta D_{i}$$

(10) $$\text{Size}-\text{mediated}\;\text{complementarity}\;\text{effects}=\sum_{i=1}^{N}\frac{\bar{M}}{{2M}_{i}}\left(D_{O,i}+D_{E,i}\right)\Delta W_{i}$$

(11) $$\text{Size}-\text{mediated}\;\text{selection}\;\text{effects}=\sum_{i=1}^{N}\frac{M_{i}-\bar{M}}{{2M}_{i}}\left(D_{O,i}+D_{E,i}\right)\Delta W_{i}$$

where $M_{i}$ is the observed biomass of invasive species *i* in monocultures, $W_{O,i}$ is the observed mean individual biomass of invasive species *i* in the mixture, $W_{E,i}$ is the expected mean individual biomass of invasive species *i* in the mixture (based on its observed individual biomass in monocultures), $\Delta W_{i}$ is the difference between $W_{O,i}$ and $W_{E,i}$; $D_{O,i}$ is the observed realised abundance of invasive species *i* in the mixture; $D_{E,i}$ is expected realised abundance of invasive species *i* in the mixture (calculated as the product of its initial planting proportion and the observed realised abundance), $\Delta D_{i}$ is the difference between $D_{O,i}$ and $D_{E,i}$.

### 2.7 Data analysis

We used one-sample t-tests with false discovery rate (FDR) correction to evaluate whether Dᵢ and DOE differed significantly from zero. Linear mixed-effect models (LMMs) were fitted using the *lmer* function in *lme4* packages (Bates et al., 2015) to test the effects of invasive species identity, abundance, and their interaction on D_i_ within each invasive species combination, with block included as a random factor. To evaluate the effects of invasive plant richness, abundance and their interaction on DOE, LMMs were also fitted with block as a random factor. Significance was assessed with Type II χ^2^ test via *car::Anova* (Fox and Weisberg, 2019), followed by post hoc pairwise t-tests with FDR adjustment (Benjamini-Hochberg method, *α* = 0.05).

LMMs were also applied to test the effects of richness and abundance on invasion success, invasive biomass, native biomass, total biomass, and biodiversity effects (net diversity, complementarity, selection, and density-mediated and size-mediated complementarity and selection effects). One-sample t-tests with FDR correction were then used to determine whether these biodiversity effects significantly deviated from zero. Ordinary least-squares regressions were used to relate invasion success index, invasive biomass, and native biomass to net diversity and complementarity effects. Polynomial regressions were used to describe the relation of invasion success index, invasive biomass, and native biomass to selection effects. In addition, we performed linear regression analyses to examine the relationships between invasive biomass and native biomass across all richness–abundance treatments, as well as under multi-species co-invasion scenarios. We also assessed the relationships between overall selection effects and their density- and size-mediated components. Independent-samples t-tests with FDR correction were used to compare observed and expected values of invasive biomass per species, realised abundance, and individual biomass of invasive plants. To explore whether environmental changes mediated invasion outcomes, we fitted LMMs with soil- and litter-related variables as response variables and richness × abundance as predictors, and analyzed the Pearson’s correlations between environmental factors and invasion success index, invasive biomass, and native biomass. We verified the assumptions of normality and homogeneity of variance before conducting statistical analyses. When these assumptions were violated, data were log-transformed to improve normality and stabilize variances.

## 3 Results

**Fig. 2:**
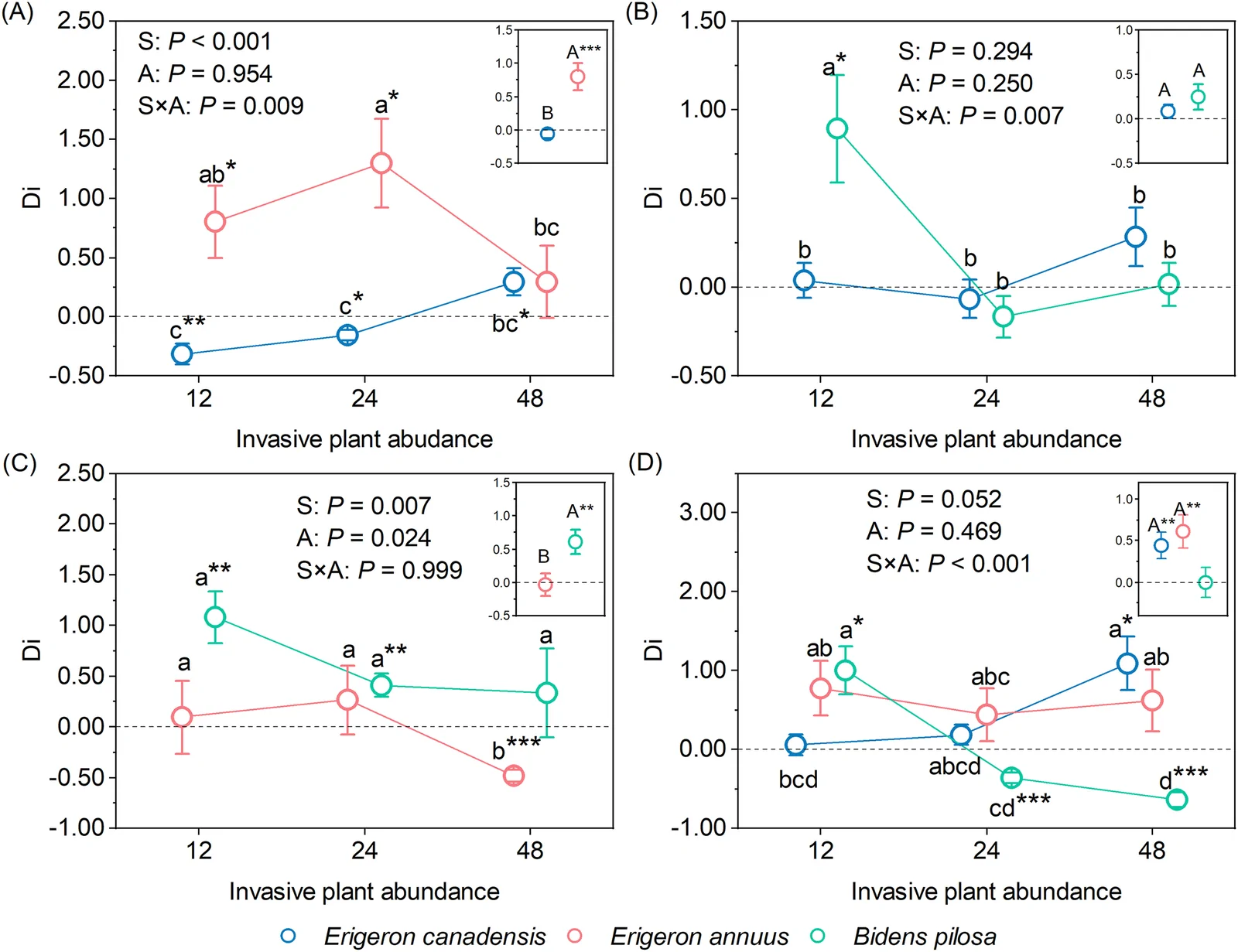
Mean (±SE) biomass deviation of invasive plant species (D_i_) in combinations of *Erigeron canadensis*-*E. annuus* (A), *E. canadensis*-*Bidens pilosa* (B), *E. annuus*-*B. pilosa* (C), and *E. canadensis*-*E. annuus*-*B. pilosa* (D). Blue, red, and green open circles represent *E. canadensis*, *E. annuus*, and *B. pilosa*, respectively. Insets report linear mixed model outcomes for species (S), abundance (A), and their interaction (S × A) effects (*E*. *canadensis*-*E*. *annuus*, *E. canadensis*-*B*. *pilosa*, *E. annuus*-*B. pilosa*: *n* = 48; *E. canadensis*-*E. annuus*-*B. pilosa*: *n* = 72). Box in each panel showed the mean (±SE) D_i_ of invasive species across abundance levels. Asterisks indicate significant differences between D_i_ and zero (one-sample t-tests, false discovery rate [FDR]-corrected): ∗∗∗*P* < 0.001, ∗∗ 0.001 < *P* < 0.01, ∗ 0.01 < *P* < 0.05. Capital letters denote significant differences among invasive plant species, and lowercase letters distinguish differences among all species–abundance combinations (FDR-corrected pairwise t-tests, *α* = 0.05).

Overall, the biomass deviation of invasive plant species (D_i_) values were either significantly positive or not significantly different from zero, indicating positive or neutral interactions among invaders (Fig. 2). In the *Erigeron canadensis*-*E. annuus* combination, the overall D_i_ values for *E. annuus* were positive and higher than those for *E. canadensis* (Fig. 2A). In the *E. annuus*-*Bidens pilosa* combination, D_i_ values for *B. pilosa* were positive and exceeded those of *E. annuus* (Fig. 2C). In three species combination, *E. canadensis* and *E. annuus* showed positive D_i_ values, whereas *B. pilosa* showed non-significant values (Fig. 2D).

**Fig. 3:**
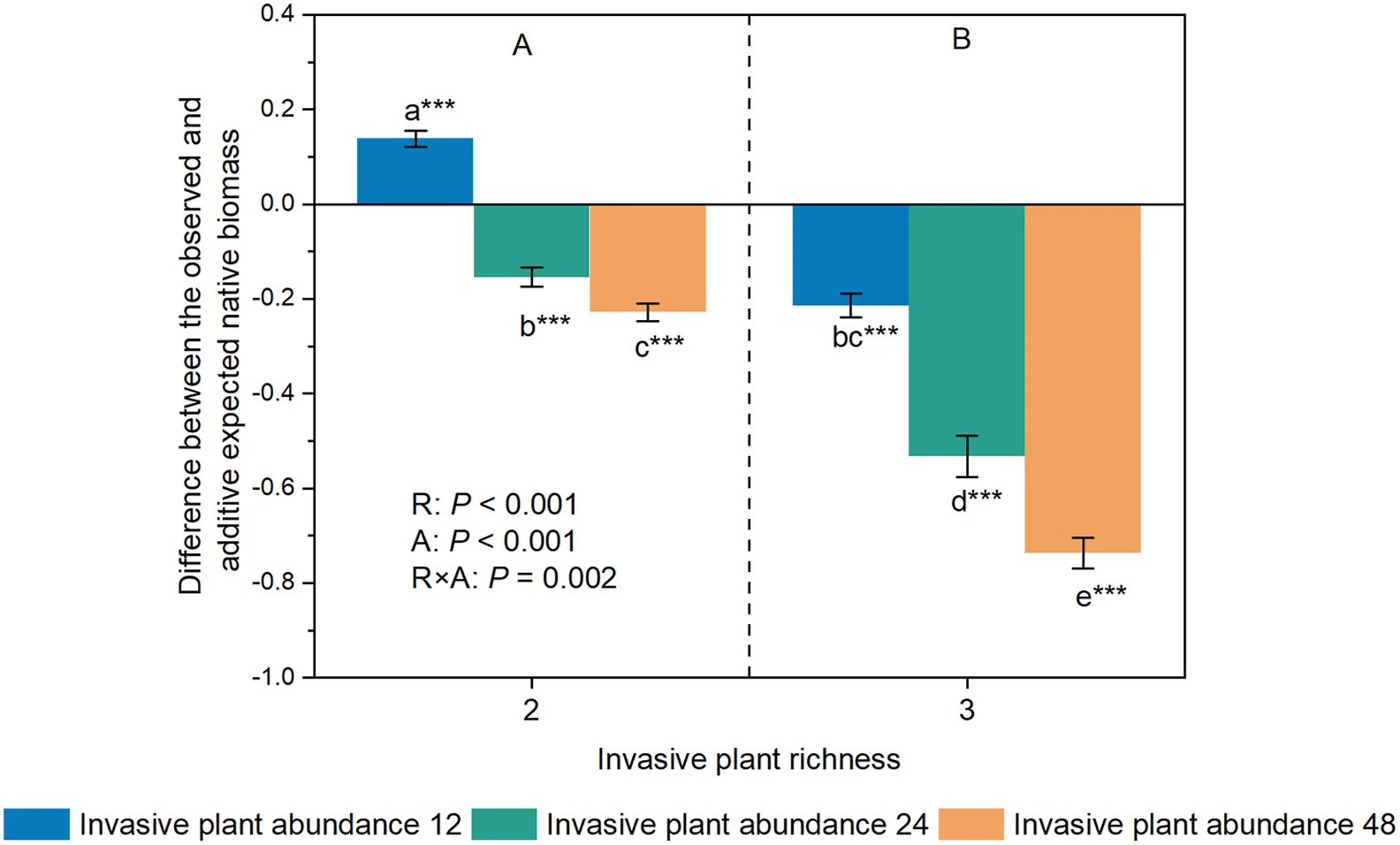
Effects of invasive plant richness and abundance on the difference between the observed and additive expected native biomass (DOE). Bars represent mean ± SE. Bar colors indicate abundance levels: blue (12 invaders), green (24), yellow (48). Insets report linear mixed model outcomes for richness (R), abundance (A), and their interaction (R × A) effects. Asterisks indicate significant differences between DOE and zero (one-sample t-tests, false discovery rate [FDR]-corrected): ∗∗∗*P* < 0.001, ∗∗ 0.001 < *P* < 0.01, ∗ 0.01 < *P* < 0.05. Capital letters denote significant differences among richness levels, whereas lowercase letters indicate differences among all richness–abundance combinations (FDR-corrected pairwise t-tests, *α* = 0.05).

Native biomass deviated significantly from additive expectations (DOE ≠ 0), indicating non-additive effects of invasive species interactions on native plants (Fig. 3). Except richness 2-abundance 12 treatment, the observed and additive expected native biomass had different signs, indicating the non-additive effects were reversal. Both invasive plant richness and abundance exerted significant negative effects on DOE, with abundance amplifying the suppressive influence of richness (Fig. 3).

**Fig. 4:**
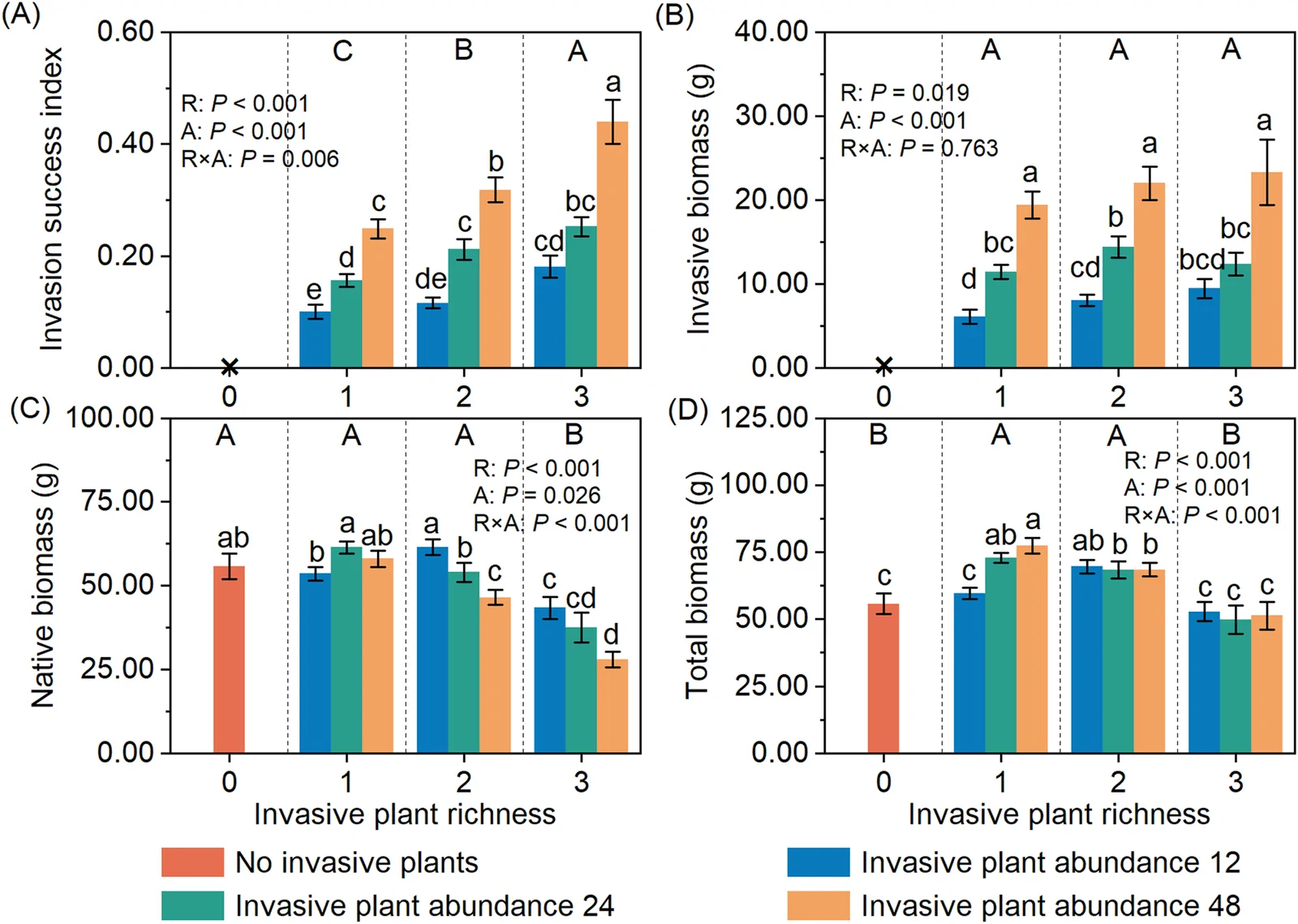
Effects of invasive plant richness and abundance on (A) invasion success index, (B) invasive biomass, (C) native biomass, and (D) total biomass. Bars represent mean ± SE. Bar colors indicate abundance levels: orange (0 invaders), blue (12), green (24), yellow (48). Insets report linear mixed model outcomes for richness (R), abundance (A), and their interaction (R × A) effects (invasive success index and invasive biomass: *n* = 168; native and total biomass: *n* = 176). Capital letters denote significant differences among richness levels, whereas lowercase letters indicate differences among all richness–abundance combinations (false discovery rate [FDR]-corrected pairwise t-tests, *α* = 0.05).

Invasion success index and invasive biomass increased markedly with both the richness and abundance of invaders (Fig. 4A and B). Higher abundance further strengthened the positive effects of invasive plant richness on invasion success (invasive plant richness × abundance: *P* = 0.006, Fig. 4A). Native biomass remained relatively stable under low-to-moderate invasion levels but declined sharply at higher richness and abundance (Fig. 4C). A significant synergistic interaction (*P* < 0.001) indicated that increasing invader abundance magnified the negative effect of richness on native biomass (Fig. 4C). Native biomass was negatively correlated with invasive biomass (Fig. S2). Total biomass exhibited a unimodal response to invasive plant richness, peaking at intermediate levels before declining (*P* < 0.001), while increasing steadily with higher invader abundance (*P* < 0.001, Fig. 4D). Invasive plant richness attenuated the positive effect of abundance on total biomass (richness × abundance: *P* < 0.001, Fig. 4D).

**Fig. 5:**
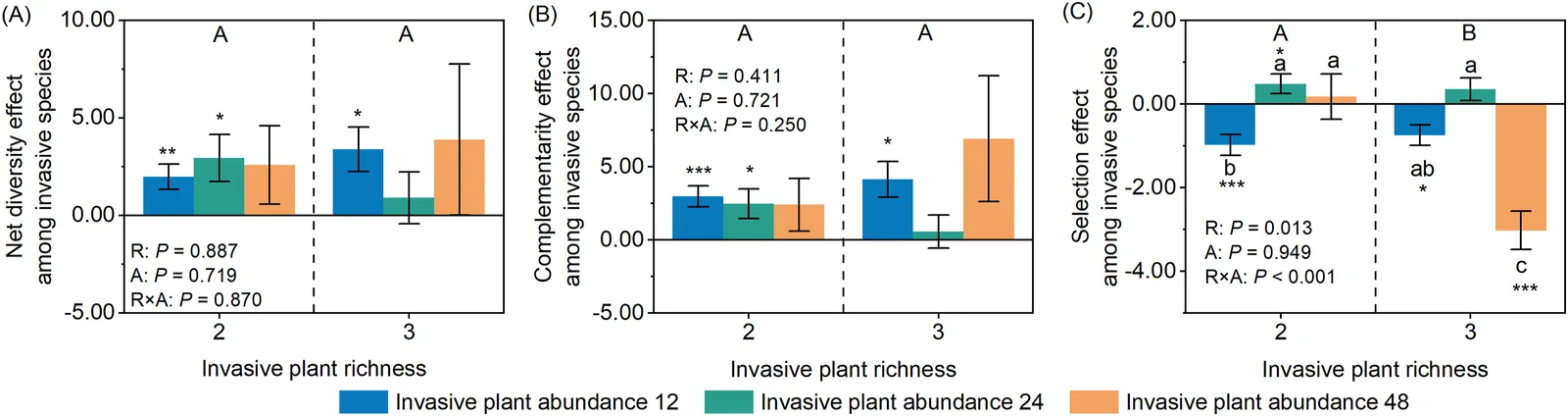
Effects of invasive plant richness and abundance on (A) net diversity effect, (B) complementarity effect, and (C) selection effect among invasive species. Bars represent mean ± SE. Bar colors indicate abundance levels: blue (12 invaders), green (24), yellow (48). Insets report linear mixed model outcomes for richness (R), abundance (A), and their interaction (R × A) effects (*n* = 96). Asterisks indicate significant deviations from zero (one-sample t-tests, false discovery rate [FDR]-corrected): ∗∗∗*P* < 0.001, ∗∗ 0.001 < *P* < 0.01, ∗ 0.01 < *P* < 0.05. Capital letters denote significant differences among richness levels, whereas lowercase letters indicate differences among all richness–abundance combinations (FDR-corrected pairwise t-tests, *α* = 0.05).

**Fig. 6:**
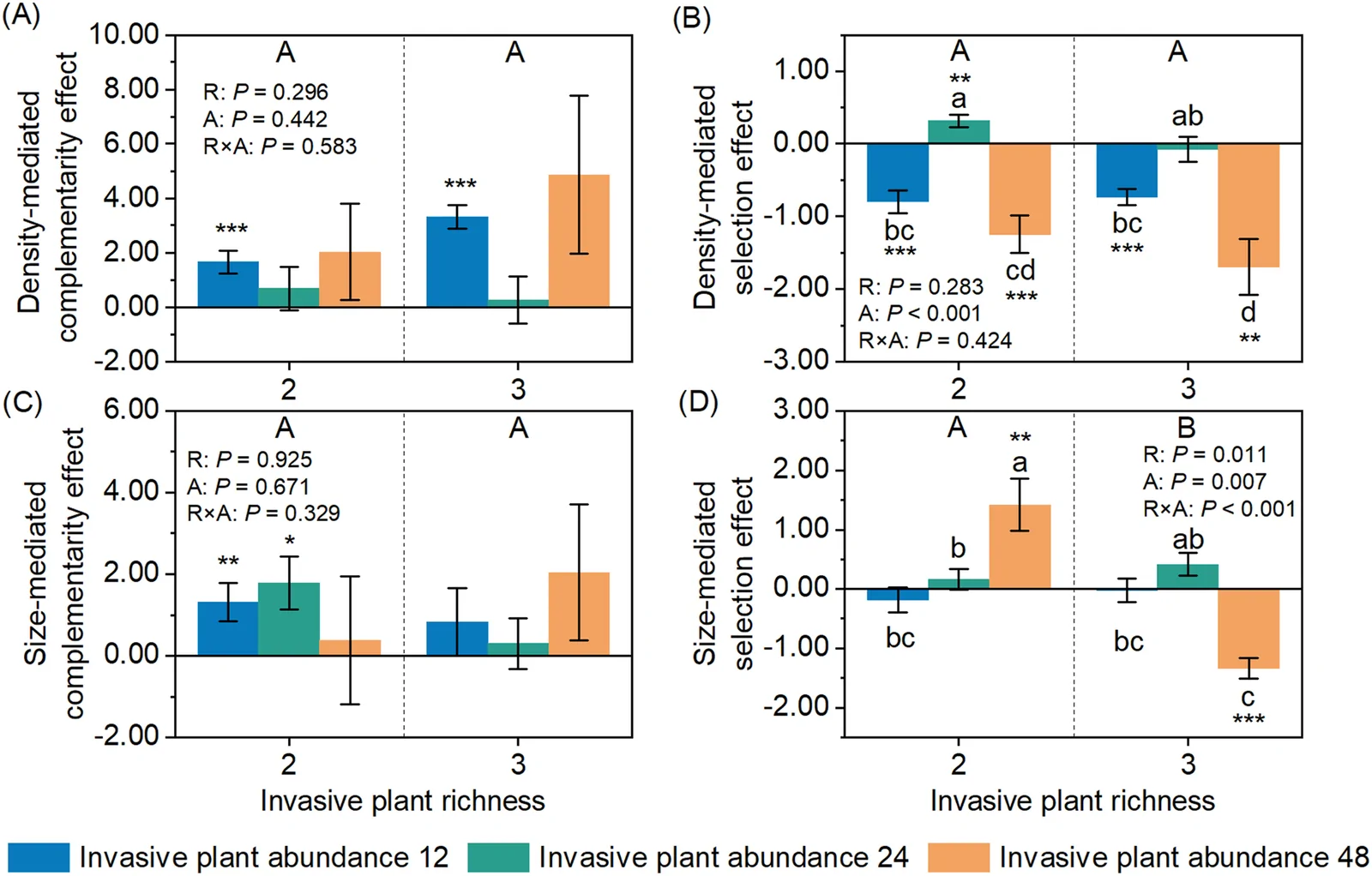
Effects of invasive plant richness and abundance on (A) density-mediated complementarity effect, (B) density-mediated selection effect, (C) size-mediated complementarity effect, and (D) size-mediated size effect among invasive species. Bars represent mean ± SE. Bar colors indicate abundance levels: blue (12 invaders), green (24), yellow (48). Insets report linear mixed model outcomes for richness (R), abundance (A), and their interaction (R × A) effects (*n* = 96). Asterisks indicate significant deviations from zero (one-sample t-tests, false discovery rate [FDR]-corrected): ∗∗∗*P* < 0.001, ∗∗ 0.001 < *P* < 0.01, ∗ 0.01 < *P* < 0.05. Capital letters denote significant differences among richness levels, whereas lowercase letters indicate differences among all richness–abundance combinations (FDR-corrected pairwise t-tests, *α* = 0.05).

Invasive plant richness, abundance, and their interaction exerted no significant effects on net diversity (Fig. 5A), complementarity (Fig. 5B), density-mediated (Fig. 6A), and size-mediated complementarity effects (Fig. 6C) among invasive species. In contrast, invasive plant richness significantly reduced selection effects (Fig. 5C). At invasive plant abundance 48, selection effects under richness 3 were significantly lower than those under richness 2. For richness 2, selection effects increased with abundance, whereas for richness 3, they initially increased and then declined. Elevated invader abundance significantly altered the relationship between richness and selection effects, rendering the relationship negative (*P* < 0.001, Fig. 5C). The density-mediated selection effect first increased and then decreased with rising abundance (*P* < 0.001, Fig. 6B). Invasive plant richness decreased size-mediated selection effects, whereas abundance enhanced them (Fig. 6D). With increasing abundance, the relationship between richness and size-mediated selection effects became negative (*P* < 0.001, Fig. 6D). Both density-mediated and size-mediated selection effects were positively correlated with overall selection effects, with a higher R^2^ for size-mediated effects (Fig. S3). The patterns of size-mediated selection effects were consistent with those of overall selection effects. Therefore, selection effects were mainly driven by size-mediated selection effects.

At an abundance of 12, selection effects were non-significant in the *Erigeron canadensis*-*E. annuus* combination, whereas in other combinations they were negative (Fig. S4). The biomass of *B. pilosa* was lower than its mean monoculture biomass but increased in mixture, leading to negative selection effects (Fig. S4). These negative selection effects were primarily driven by realised abundance (Figs. S5D, G, J). At abundance 48, the biomass of *E. canadensis*—which was lower than its mean monoculture biomass—increased in the three-species mixture, while that of *B. pilosa*—which exceeded its monoculture mean—decreased in mixture, again resulting in negative selection effects (Fig. S4O and P). These negative selection effects in the *E. canadensis*-*E. annuus*-*B. pilosa* mixture were mainly driven by realised abundance and individual invasive biomass (Fig. S5L).

**Fig. 7:**
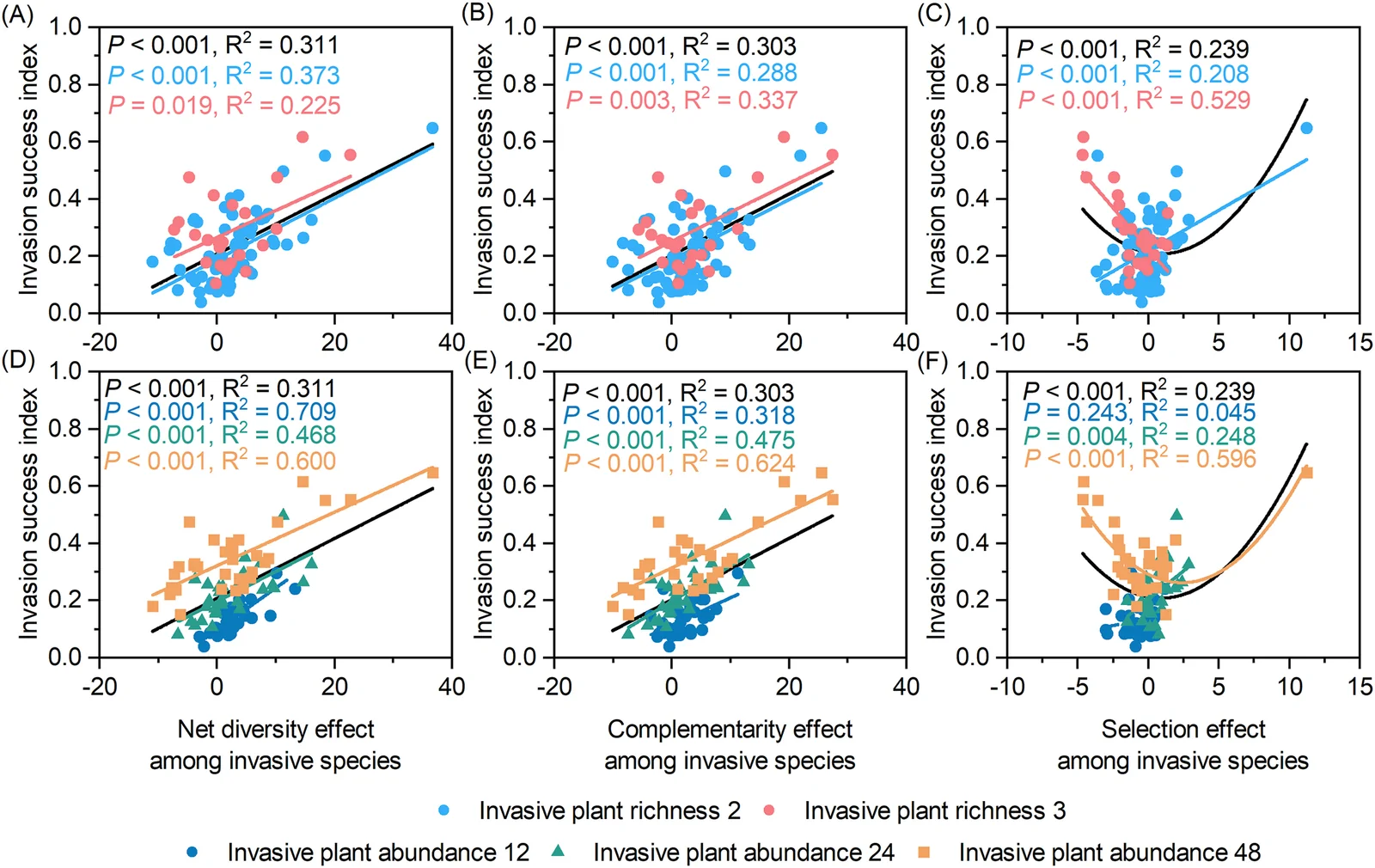
Relationships between the invasion success index and (A) net diversity effect, (B) complementarity effect, and (C) selection effect among invasive species across invasive plant richness treatments, and between the invasion success index and (D) net diversity effect, (E) complementarity effect, and (F) selection effect among invasive species across invasive plant abundance treatments. Insets present statistical significance and R^2^ from linear or polynomial regression. Solid lines indicate significant relationships (*P* < 0.05); dashed lines indicate non-significant ones (*P* > 0.05). Black lines represent overall relationship across all treatments. Light blue and red circles denote invasive plant richness levels of 2 (*n* = 72) and 3 (*n* = 24), respectively. Symbols and colors indicate abundance levels: blue circles (12, *n* = 32), green triangles (24, *n* = 32), and yellow squares (48, *n* = 32).

Complementarity and net diversity effects were positively correlated with invasion success index (Fig. 7A, B, D, E) and invasive biomass (Fig. S6A and B) across different invasive plant richness and abundance treatments, whereas native biomass showed no correlations with complementarity or net diversity effects (Fig. S6D and E). As selection effects increased, both invasion success index (Fig. 7C and F) and invasive biomass (Fig. S6C) first decreased and then increased, while native biomass showed the opposite pattern, increasing initially and then declining (Fig. S6F), and the inflection points were all near the selection effects of zero. Under richness 2, selection effects were positively associated with invasion success index, while under richness 3 the relationship was negative (Fig. 7B).

To further explore the soil mechanisms underlying invasion success, we examined how invasive plant richness and abundance affected soil properties and microbial activity. Invasive plant richness significantly altered several environmental variables, whereas abundance had limited influence. Higher invader richness increased soil total nitrogen (Fig. S7A) but reduced soil NO_3_^-^–N, NH_4_^+^–N, and microbial biomass carbon (Fig. S7B, C, J). The strongest positive effects of invasive richness on β-glucosaminidase activity and litter loss occurred at intermediate richness levels (Fig. S7D and J). Invasive biomass was negatively associated with microbial biomass carbon; similarly, the invasion success index was negatively related to both microbial biomass carbon and litter loss (Fig. S8). In contrast, native biomass was positively correlated with microbial biomass carbon, β-glucosidase activity, and litter loss (Fig. S8).

## 4 Discussion

While numerous studies have examined facilitative or competitive interactions between pairs of invasive species, few have simultaneously investigated how multiple invaders interact and collectively influence native communities under intensified co-invasion (Kuebbing and Nunez, 2015; Braga et al., 2020; Vujanovic et al., 2022; Lone et al., 2024). Moreover, the combined effects of invasive plant richness and abundance on invasion success remain poorly understood. Our study advances this understanding by providing experimental evidence that interactions among multiple invaders can generate non-additive effects on native communities and modulate invasion success, supporting invasional meltdown hypothesis. Specifically, increasing invasive plant richness altered selection effects among invaders, thereby increasing invasive biomass and intensifying suppression of native species. In addition, increasing invader abundance further promoted invasion success and caused selection effect to decline progressively along the richness gradient. Collectively, these findings reveal a synergistic mechanism through which invasive plant richness and abundance jointly facilitated invasion success under multi-species co-invasion.

Although competitive and neutral interactions are typically predominant among invasive species (Kuebbing and Nunez, 2015, 2016; Ferenc and Sheppard, 2020; Ren et al., 2023), our results demonstrate that facilitative and neutral effects co-occurred, leading to enhanced invasive biomass with increasing invasive plant richness. Previous studies have shown that invasive species can promote co-occurring invaders by improving soil nutrient availability (Von Holle et al., 2006; Oschrin and Reynolds, 2019). In our study, although invasive plant richness elevated total soil nitrogen, it concurrently reduced soil NO_3_^-^–N and NH_4_^+^–N concentrations. These patterns likely reflect altered nitrogen cycling and increased nutrient uptake by a more diverse invasive assemblage. However, invasive biomass showed no significant relationship with total or available nitrogen, suggesting that enhanced invasion success was not directly driven by soil nutrient availability. Instead, selection effects primarily explained variation in invasive biomass. The relationship between selection effects and invasive biomass was non-linear: invasive biomass declined when selection effects were weak but increased as they became strongly negative or strongly positive, with an inflection point near zero. This pattern indicates that invasion success can be driven either by the dominance of highly productive invaders (positive selection effects) or by the enhanced performance of less-productive but competitively aggressive invaders (negative selection effects). Positive selection effects likely arose because highly competitive, dominant species contributed disproportionately to total community biomass, thereby enhancing overall invasion success (Wang et al., 2024; Xu et al., 2024, 2025). Conversely, negative selection effects emerged when species with lower-than-average monoculture biomass achieved higher biomass in mixtures (Loreau and Hector, 2001; Polley et al., 2003; Wang et al., 2025b). We observed that positive interactions among invaders facilitated the growth of less-productive but strongly competitive species (Fig. 2 and Fig. S4). Although the dominance of highly productive invasive species decreased when multi-species co-invaded, the facilitative growth of less-productive species can stabilise community-level biomass, collectively resulting in higher-than-expected invasive biomass and stronger suppression of native plants (Oschrin and Reynolds, 2019). To further elucidate these dynamics, we partitioned selection effects into density-mediated and size-mediated components (Tatsumi and Loreau, 2023) and found that richness primarily influenced the size-mediated component rather than the density-mediated one. This suggests that changes in individual growth (i.e., morphological or physiological plasticity), rather than differences in survival, underlie the richness–invasion relationship.

Consistent with these findings, native biomass was largely unaffected by monospecific invasions but declined sharply under multispecies co-invasion. During the early stages of invasion, a single invasive species may occupy unfilled niche space and thereby avoid intense competition with natives (MacArthur, 1970; MacDougall et al., 2009). However, when multiple species co-invaded, interactions among invasive species imposed non-additive negative effects on native communities, which intensified with increasing invasive plant richness (Fig. 3). This likely occurred because strong positive or negative selection effects enhanced invader growth, progressively reducing the availability of unoccupied ecological niches, amplifying the suppression of native biomass, and ultimately promoting invasion success. The co-occurrence of facilitative and neutral interactions among invasive species, the intensified non-additive suppression of native biomass, and the enhanced invasion success with greater invader richness collectively provide strong empirical support for the invasional meltdown hypothesis (Simberloff and Von Holle, 1999). Our findings are consistent with those of Wang et al. (2022), who reported that selection effects promoted invasion success as invasive plant richness increased. The limited role of complementarity effects may stem from the shared biogeographic origin and overlapping introduced ranges of the three Asteraceae invaders (*Erigeron canadensis*, *E. annuus*, and *Bidens pilosa*) in China. Their long-term co-occurrence may have facilitated partial ecological convergence through co-evolutionary processes, leading to similar resource-use strategies (Hao and Ma, 2023). The limited role of complementarity effects, together with the dominant influence of selection effects, indicates that multispecies invasion success is primarily driven by the dominance of highly productive invaders and the enhanced performance of less-productive but competitive species, rather than by niche partitioning among invaders.

Beyond selection effects, invasive plants may also release allelochemicals that inhibit native plant growth (Callaway and Ridenour, 2004; Wang et al., 2025a). In our study, increasing invasive plant richness reduced soil microbial biomass carbon and litter loss. Both microbial biomass carbon and litter loss were negatively correlated with invasion success but positively correlated with native biomass, while microbial biomass carbon was negatively associated with invasive biomass. These patterns suggest that increasing invader richness may facilitate invasion through allelopathic effects that modify soil microbial community composition and impair microbial functioning (Guo et al., 2023b; Colautti and Antunes, 2025). Such alterations can disrupt beneficial plant–microbe interactions, weaken native plant performance, and create positive plant–soil feedbacks that favour further invasion.

We also found increasing invasive plant abundance enhanced invasion success, consistent with studies demonstrating that propagule pressure promotes establishment and persistence of invasive species (Petri and Ibañez, 2023; Munné-Bosch, 2024). Elevated propagule pressure can enhance population fitness (Lockwood et al., 2005; Simberloff, 2009), thereby overcoming Allee effects—the reduced growth and survival rates observed at low population densities (Drake and Lodge, 2006). Greater invader abundance increased invasive biomass, collectively conferring competitive advantages that intensified suppression of native plants (Lockwood et al., 2005; Simberloff, 2009; Stringham and Lockwood, 2021). Notably, our results revealed that elevated invasive abundance amplified the suppressive influence of richness on native biomass while simultaneously strengthening its facilitative effect on invasion success. Higher abundance also rendered the relationship between selection effects and richness negative, thereby enhancing invasive biomass and enabling invaders to occupy previously available niche space and pre-empt resources and habitat from native species. Consequently, positive interactions among invasive plants, when combined with high abundance, generated synergistic effects that accelerated competitive exclusion and diminished native dominance (Carboni et al., 2021; Hart, 2023). Collectively, these findings suggest that the severity of multispecies co-invasion may escalate under future scenarios of increasing propagule pressure.

Species within the Asteraceae family exhibit a high propensity for co-invasion (Chen et al., 2013), and this family encompasses the greatest number of invasive species worldwide (Pyšek et al., 2017; Yang et al., 2025). Our findings of facilitative interactions among invaders should therefore be interpreted in the context that all three species examined belong to the Asteraceae family, which likely share similar ecological niches and functional traits—such as comparable resource-use strategies, strong competitive advantages over native species, and allelopathic potential—that may facilitate their co-invasion. Consequently, the generality of these findings to invasions involving more functionally divergent groups (e.g., grasses, forbs, or legumes) remains to be tested. Future studies should integrate plant functional trait measurements and phylogenetic relatedness to disentangle the underlying mechanisms driving the synergistic effects of invader richness and abundance.

## 5 Conclusions

In summary, this study simultaneously examined facilitative and neutral interactions among three Asteraceae invaders and their non-additive negative effects on native biomass, which collectively enhanced invasion success, providing empirical support for the invasional meltdown hypothesis. Invasive plant richness enhanced co-invader performance through selection effects—mainly mediated by size-dependent mechanisms—thereby promoting collective establishment and dominance while intensifying suppression of native species. Meanwhile, multispecies invasion success was associated with shifts in soil microbial processes rather than increased soil nitrogen availability. These findings elucidate a mechanism through which invasive plant richness promotes invasion success. Furthermore, increasing invasive plant abundance not only facilitated invasion success but also magnified the positive influence of richness on invasion outcomes, likely by reinforcing negative selection effects among co-occurring invaders. From a management perspective, our results highlight that mitigating multispecies co-invasion requires strategies targeting both species richness and abundance. Specifically, prioritising the removal of low-abundance, easily controllable invaders may effectively reduce community-level richness and disrupt the synergistic relationship between invader richness and abundance. Following co-invader removal, restoring diverse native plant communities is critical to resisting secondary invasions and to maintaining ecosystem stability and functioning.

## CRediT authorship contribution statement

**Yi Hu**: Writing – original draft, Visualization, Methodology, Investigation, Formal analysis, Conceptualization. **Xiao Guo**: Writing – review & editing, Funding acquisition, Formal analysis, Conceptualization. **Zhenwei Xu**: Writing – review & editing, Investigation, Formal analysis, Conceptualization. **Jingfeng Wang**: Writing – review & editing, Investigation. **Ruiqi Sun**: Writing – review & editing, Formal analysis. **Mingyan Li**: Writing – review & editing. **Pengcheng Zhu**: Writing – review & editing, Funding acquisition. **Karin M. Kettenring**: Writing – review & editing. **Hana Skálová**;: Writing – review & editing. **Weihua Guo**: Writing – review & editing, Funding acquisition, Conceptualization.

## Data availability statement

The data supporting the findings of this study are available in Figshare Database: 10.6084/m9.figshare.25239913.

## Declaration of competing interest

The authors declare that they have no known competing financial interests or personal relationships that could have appeared to influence the work reported in this paper.
