# Supplementary material for: Invasive plant richness and abundance synergistically amplify co-invasion success: Experimental evidence for invasional meltdown
Source: Plant Divers. 2026 Mar 11;48(5):915–26. doi: 10.1016/j.pld.2026.03.004 (PMC13615784; doi:10.1016/j.pld.2026.03.004)
Supplement: Supplementary file 1 [file mmc1.docx]

Invasive plant richness and abundance synergistically amplify co-invasion success: Experimental evidence for invasional meltdown

**Supporting methods**

**Study site**

A mesocosm experiment was carried out in Ma Jia Bai Miao Village (36°22′ N, 120°36′ E), located in Qingdao, Shandong Province, China, during the period from June to November 2021. The research was conducted in a greenhouse (800 m^2^) with an average temperature of 26.3 °C and an average humidity of 75.3% during the experiment period. These favorable growing conditions were upheld through the strategic use of plastic films to provide shading and proper ventilation. Additionally, the intensity of the light within the greenhouse was measured to be 80% of that of full sunlight.

**Measurement**

To assess litter decomposition, nylon litter bags containing 10 g of reeds were placed in the 5 cm-depth soil of each mesocosm at the beginning of the experiment. The difference in weight between the litter bags at the beginning of the experiment and the harvest served as an indicator of litter decomposition. Litter bags, initially placed within the soil, were later excavated, and the remaining litter underwent oven-drying at 80 ^o^C before being weighed. The difference in weight between the litter bags at the beginning of the experiment and the harvest served as an indicator of litter decomposition. Soil total nitrogen was quantified using the Kjeldahl method (Bremner, 1960). To assess soil nitrogen availability, fresh soil samples were extracted using calcium chloride, and soil NH_4_^+^-N and NO_3_^⁻^-N were determined using the indophenol-blue colorimetric method (Searle, 1984) and the hydrazine sulfate reduction method (Kamphake et al., 1967), respectively. Soil β-glucosaminidase activity, β-glucosidase activity, and acid phosphatase activity were measured by quantifying the amount of ρ-nitrophenol produced. Soil β-glucosaminidase activity associated with soil nitrogen cycle was determined using the method described by Eivazi and Tabatabai (1988), β-glucosidase activity related to soil carbon cycle was assessed following the technique outlined by Parham and Deng (2000), and acid phosphatase activity connected with soil phosphorus was measured using the approach developed by Dick (2011). Soil phosphorus was determined using the Molybdenum antimony-D-isoascorbic-acid colorimetry method (Zhang et al., 2013), while soil organic carbon content was quantified using the potassium dichromate oxidation-external heating method (Nelson and Sommers, 1983). We quantified microbial biomass carbon using the arginine induction method (Alef and Kleiner, 1986).

**Supporting figures**

**Fig. S1** A detailed schematic illustration of the experimental design showing the planting pattern of invasive and native plants.

**Fig. S2** Relationships between invasive biomass and native biomass across all richness treatments (A, *n* = 168), and under multi-species invasion treatments only (B, *n* = 96). Insets present statistical significance and R^2^ based on linear regression. Solid lines mean significant (*P* < 0.05) relationships and dashed lines represent non-significant (*P* > 0.05) relationships.

**Fig. S3** Relationships between selection effect among invasive species and density-mediated selection effect (A), and size-mediated selection effect (B). Insets present statistical significance and R^2^ based on linear regression (*n* = 96). Solid lines mean significant (*P* < 0.05) relationships and dashed lines represent non-significant (*P* > 0.05) relationships.

**Fig. S4** Mean (± SE) expected and observed invasive biomass, and selection effect among invasive species under different invasive plant abundance treatments. The four horizontal panels represent species combinations: *Erigeron canadensis*-*E. annuus*, *E. canadensis*-*Bidens pilosa*, *E. annuus*-*B. pilosa*, and *E. canadensis*-*E. annuus*-*B. pilosa*. Blue, red, and green filled circles represent expected values of *E. canadensis*, *E. annuus*, and *B. pilosa*, respectively, whereas blue, red, and green open circles represent their corresponding observed values. The three vertical panels correspond to invasive plant abundance levels of 12 (A, E, I, M), 24 (B, F, J, N), and 48 (C, G, K, O) individuals, showing expected and observed invasive biomass for each combination. The far-right column (D, H, L, P) shows the corresponding selection effects, where bar colors indicate abundance levels: blue (12 invaders), green (24), yellow (48). *P*-values indicate whether differences in expected or observed invasive biomass exist among species. Lowercase letters denote differences among expected biomass values across species based on false discovery rate (FDR)-corrected pairwise t-tests. Asterisks following lowercase letters indicate significant differences from expected biomass and mean values (dashed horizontal line) based on one-sample t-tests corrected for FDR: *** *P* < 0.001, ** 0.001 < *P* < 0.01, * 0.01 < *P* < 0.05. Capital letters denote differences among observed biomass values across species (FDR-corrected pairwise t-tests, α = 0.05). Asterisks on the connecting lines between expected and observed biomass represent the significant differences between them. In the far-right panels, lowercase letters indicate differences in selection effects across abundance treatments (FDR-corrected pairwise t-tests), and asterisks denote significant deviations of selection effects from zero (one-sample t-tests, FDR-corrected).

**Fig. S5** Mean (± SE) expected and observed values of realised abundance and individual biomass of invasive plants under different invasive plant abundance treatments. The four horizontal panels represent species combinations: *Erigeron canadensis*-*E. annuus*, *E. canadensis*-*Bidens pilosa*, *E. annuus*-*B. pilosa*, and *E. canadensis*-*E. annuus*-*B. pilosa*. Blue, red, and green filled circles represent expected values of *E. canadensis*, *E. annuus*, and *B. pilosa*, respectively, whereas blue, red, and green open circles represent their corresponding observed values. The three vertical panels correspond to invasive plant abundance levels of 12 (A, D, G, J), 24 (B, E, H, K), and 48 (C, F, I, L) individuals, showing the expected and observed values of realised abundance and individual biomass for each combination. Asterisks on the connecting line along the x-axis indicate significant differences between expected and observed realised abundance (one-sample t-tests, false discovery rate [FDR]-corrected). Asterisks on the connecting line along the y-axis indicate significant differences between expected and observed individual biomass (one-sample t-tests, FDR-corrected): *** *P* < 0.001, ** 0.001 < *P* < 0.01, * 0.01 < *P* < 0.05, ns *P* > 0.05.

**Fig. S6** Relationships between invasive biomass and (A) net diversity effect, (B) complementarity effect, and (C) selection effect among invasive species; and relationships between native biomass and (D) net diversity effect, (E) complementarity effect, and (F) selection effect among invasive species. Insets indicate statistical significance and R^2^ based on linear or polynomial regression (*n* = 96). Solid lines represent significant relationships (*P* < 0.05), whereas dashed lines indicate non-significant relationships (*P* > 0.05).

**Fig. S7** Mean (± SE) soil total nitrogen (A), soil NO_3_⁻-N (B), soil NH_4_^+^-N (C), β-glycosaminidase activity (D), soil organic carbon (E), microbial biomass carbon (F), β-glucosidase activity (G), soil total phosphorus (H), acid phosphatase activity (I), and litter loss (J) in response to the effects of invasive plant richness and abundance. Bar colors denote abundance levels: orange (0 invaders), blue (12), green (24), yellow (48). Statistical insets report linear mixed model outcomes for richness (R), abundance (A), and their interaction (R×A) effects (n = 176). Capital letters indicate significant richness-level differences (false discovery rate [FDR]-corrected pairwise t-tests, α = 0.05), lowercase letters distinguish differences across all richness-abundance combinations.

**Fig. S8** Pearson’s correlations between environmental factors and native biomass, invasive biomass, and invasion success index. Significant Pearson’s correlation coefficients are marked. *** *P* < 0.001, ** 0.001 < *P* < 0.01, * 0.01 < *P* < 0.05. Color gradient and square size mean the correlation coefficient direction and magnitude. Red and blue represent the correlation coefficient is 1 and -1, respectively.

**Supporting Tables**

**Table S1** List of the eight native species and three invasive species.

| Status | Family | Life form | Scientific name | Native range of invasive species |
| --- | --- | --- | --- | --- |
| Invasive | Asteraceae | annual | *Erigeron canadensis* | North America |
| Invasive | Asteraceae | annual | *Erigeron annuus* | North America |
| Invasive | Asteraceae | annual | *Bidens Pilosa* | Tropical America |
| Native | Amaranthaceae | annual | *Suaeda glauca* |  |
| Native | Amaranthaceae | annual | *Suaeda salsa* |  |
| Native | Apocynaceae | perennial | *Apocynum venetum* |  |
| Native | Plumbaginaceae | perennial | *Limonium bicolor* |  |
| Native | Asteraceae | perennial | *Artemisia capillaris* |  |
| Native | Fabaceae | annual | *Glycine soja* |  |
| Native | Poaceae | perennial | *Phragmites australis* |  |
| Native | Poaceae | annual | *Setaria viridis* |  |

**Table S2** Experimental design list

| Richness | Invasive species | Abundance | Diversity |
| --- | --- | --- | --- |
| 0 | No | 0 | 2.079 |
| 1 | *Erigeron canadensis* | 12 | 2.164 |
| 1 | *E. canadensis* | 24 | 2.023 |
| 1 | *E. canadensis* | 48 | 1.733 |
| 1 | *E. annuus* | 12 | 2.164 |
| 1 | *E. annuus* | 24 | 2.023 |
| 1 | *E. annuus* | 48 | 1.733 |
| 1 | *Bidens Pilosa* | 12 | 2.164 |
| 1 | *B. pilosa* | 24 | 2.023 |
| 1 | *B. pilosa* | 48 | 1.733 |
| 2 | *E. canadensis* and *E. annuus* | 12 | 2.303 |
| 2 | *E. canadensis* and *E. annuus* | 24 | 2.254 |
| 2 | *E. canadensis* and *E. annuus* | 48 | 2.079 |
| 2 | *E. canadensis* and *B. pilosa* | 12 | 2.303 |
| 2 | *E. canadensis* and *B. pilosa* | 24 | 2.254 |
| 2 | *E. canadensis* and *B. pilosa* | 48 | 2.079 |
| 2 | *E. annuus* and *B. pilosa* | 12 | 2.303 |
| 2 | *E. annuus* and *B. pilosa* | 24 | 2.254 |
| 2 | *E. annuus* and *B. pilosa* | 48 | 2.079 |
| 3 | *E. canadensis*, *E. annuus*, and *B. pilosa* | 12 | 2.384 |
| 3 | *E. canadensis*, *E. annuus*, and *B. pilosa* | 24 | 2.389 |
| 3 | *E. canadensis*, *E. annuus*, and *B. pilosa* | 48 | 2.282 |

.

**Supporting references**

Alef, K., Kleiner, D., 1986. Arginine ammonification, a simple method to estimate microbial activity potentials in soils. Soil Biol. Biochem. 18(2): 233-235.

Bremner, J.M., 1960. Determination of nitrogen in soil by the Kjeldahl method. J. Agr. Sci. 55(1): 11-33.

Dick, R.P., 2011. Methods of soil enzymology. Soil Science Society of America, Madison.

Eivazi, F., Tabatabai, M.A., 1988. Glucosidases and galactosidases in soils. Soil Biol. Biochem. 20(5): 601-606.

Kamphake, L.J., Hannah, S.A., Cohen, J.M., 1967. Automated analysis for nitrate by hydrazine reduction. Water Res. 1(3): 205-216.

Nelson, D.W., Sommers, L.E., 1983. Total carbon, organic carbon, and organic matter, in: Page, AL (ed), *Methods of Soil Analysis*, pp. 539-579.

Parham, J.A., Deng, S.P., 2000. Detection, quantification and characterization of β-glucosaminidase activity in soil. Soil Biol. Biochem. 32(8): 1183-1190.

Searle, P.L., 1984. The Berthelot or indophenol reaction and its use in the analytical chemistry of nitrogen. A review. Analyst 109(5): 549-568.

Zhang, R., Zhou, Z.C., Luo, W.J., et al., 2013. Effects of nitrogen deposition on growth and phosphate efficiency of *Schima superba* of different provenances grown in phosphorus-barren soil. Plant Soil 370(1): 435-445.
